# Combined therapy of dabrafenib and an anti-HER2 antibody–drug conjugate for advanced BRAF-mutant melanoma

**DOI:** 10.1186/s11658-024-00555-z

**Published:** 2024-04-10

**Authors:** Weisong Li, Chao Zheng, Xi Xu, Yujie Xia, Kai Zhang, Ao Huang, Xinyu Zhang, Yong Zheng, Guofang Chen, Shuyong Zhang

**Affiliations:** 1 Department of General Surgery, First Affiliated Hospital, Gannan Medical University, Ganzhou, 341000 China; 2 Key Laboratory of Prevention and Treatment of Cardiovascular and Cerebrovascular Diseases (Ministry of Education), Gannan Medical University, 1 Hexie Road, Rongjiang New District, Ganzhou, 341000 China; 3 School of Basic Medicine, Gannan Medical University, Ganzhou, 341000 China; 4 Shanghai Key Laboratory of Maternal Fetal Medicine, Shanghai Institute of Maternal-Fetal Medicine and Gynecologic Oncology, Clinical and Translational Research Center, Shanghai First Maternity and Infant Hospital, School of Medicine, Tongji University, Shanghai, 200092 China

**Keywords:** HER2, RC48, Antibody drug conjugate, Dabrafenib, Synergetic effect, Melanoma

## Abstract

**Background:**

Melanoma is the most lethal skin cancer characterized by its high metastatic potential. In the past decade, targeted and immunotherapy have brought revolutionary survival benefits to patients with advanced and metastatic melanoma, but these treatment responses are also heterogeneous and/or do not achieve durable responses. Therefore, novel therapeutic strategies for improving outcomes remain an unmet clinical need. The aim of this study was to evaluate the therapeutic potential and underlying molecular mechanisms of RC48, a novel HER2-target antibody drug conjugate, either alone or in combination with dabrafenib, a V600-mutant BRAF inhibitor, for the treatment of advanced BRAF-mutant cutaneous melanoma.

**Methods:**

We evaluated the therapeutic efficacy of RC48, alone or in combination with dabrafenib, in BRAF-mutant cutaneous melanoma cell lines and cell-derived xenograft (CDX) models. We also conducted signaling pathways analysis and global mRNA sequencing to explore mechanisms underlying the synergistic effect of the combination therapy.

**Results:**

Our results revealed the expression of membrane-localized HER2 in melanoma cells. RC48 effectively targeted and inhibited the growth of HER2-positive human melanoma cell lines and corresponding CDX models. When used RC48 and dabrafenib synergically induced tumor regression together in human BRAF-mutant melanoma cell lines and CDX models. Mechanically, our results demonstrated that the combination therapy induced apoptosis and cell cycle arrest while suppressing cell motility in vitro. Furthermore, global RNA sequencing analysis demonstrated that the combination treatment led to the downregulation of several key signaling pathways, including the PI3K-AKT pathway, MAPK pathway, AMPK pathway, and FOXO pathway.

**Conclusion:**

These findings establish a preclinical foundation for the combined use of an anti-HER2 drug conjugate and a BRAF inhibitor in the treatment of BRAF-mutant cutaneous melanoma.

**Supplementary Information:**

The online version contains supplementary material available at 10.1186/s11658-024-00555-z.

## Introduction

Cutaneous melanoma is a highly heterogeneous and aggressive malignant tumor [1–3]. Cutaneous melanoma accounts for approximately 55,500 deaths annually, and the incidence and mortality rates of the disease vary globally, caused by inadequate medical care and a lack of access to early detection and effective therapies in many countries [4–6]. Although surgical resection remains the first choice therapeutic option for early-stage cutaneous melanoma [7, 8], some patients who were diagnosed as distant metastasis or inoperable melanoma must be implemented with systemic interventions, including chemotherapy, immunotherapy, and targeted therapy [7, 9, 10]. With the rapid development of novel therapies, the survival rate of advanced and metastatic patients has been greatly improved. Selma Ugurel et al. reported that the average 12-months survival for stage IV melanoma patients was 74.5% with BRAF plus MEK inhibitors and 71.9% for anti-PD-1 blockade [11]. A 5-year overall survival rate for metastatic melanoma has increased substantially from less than 10% to up to 40–50% based on PD-1-based treatment and targeted agents in BRAF V600-mutant melanoma [4, 12–15]. Checkmate-067 demonstrated 5-year overall survival rate of 52% for the combination of nivolumab and ipilimimab compared with nivolumab and ipilimumab monotherapies, but this exceptional survival of combination treatment was associated with 59% of patients suffering grade 3 or 4 adverse events, and many patients eventually experienced disease progression due to primary refractory disease or acquired resistance[15, 16]. In addition, some unique patients with severe autoimmune disease, organ transplants, or other complications who are not eligible for immunotherapy [17]. Thus, alternative treatment options of advanced melanoma remain challenging.

It is worth noting that BRAF mutations have been identified in 50–60% of all metastatic melanomas, with about 80–90% of these mutations involving the substitution of valine for glutamine at amino acid 600 (V600E), which is associated with a poor overall prognosis [18, 19]. Dabrafenib, a highly specific reversible inhibitor of V600-mutant BRAF kinase, has been developed to target this oncogenic mutation that drives proliferation in various tumors [20]. It binds to the active form of BRAF kinase, inducing cell cycle arrest and improving response rates and progression-free survival [21]. Currently, dabrafenib is indicated as a single agent for unresectable or metastatic melanoma with the BRAF V600E mutation and, in combination with trametinib, for advanced melanoma with BRAF mutations [20–22]. However, resistance to dabrafenib commonly occurred in patients with BRAF-mutant melanoma. Therefore, there is an immediate need to develop innovative and effective combination therapies for advanced BRAF-mutant melanoma.

Human epidermal growth factor receptor-2 (HER2) amplification and/or overexpression not only contributes to tumor occurrence and development but also serves as an important clinical indicator for treatment monitoring and prognosis. HER2 has thus emerged as an attractive target for precision medicine due to its amplification and/or overexpression in various cancers [23–26]. Antibody–drug conjugates (ADCs) serve as a potentially promising therapeutic approach for the treatment of solid tumors and hematologic malignancies. ADCs combine antibody-associated antigen specificity with cytotoxic antitumor effects, thereby maximizing efficacy and minimizing systemic toxicity [27–31]. Successful examples include Herceptin (trastuzumab), a humanized monoclonal antibody for targeting HER-2-positive breast cancer [32, 33], and ado-trastuzumab emtansine (T-DM1), a second generation ADC composed of trastuzumab conjugated to maytansoinoid emtansine (DM1). This ADC has been approved for the treatment of HER2-positive metastatic breast cancer [24]. Another promising ADC, trastuzumab deruxtecan (T-DXd), consists of trastuzumab, an enzymatically cleavable maleimide glycynglycyn-phenylalanyn-glycyn (GGFG) peptide linker, and the topoisomerase I inhibitor deruxtecan [24]. T-DXD has demonstrated efficacy against HER2-overexpression and low-expression breast cancer [34, 35]. Additionally, RC48 (disitamab vedotin), a novel humanized anti-HER2 monoclonal antibody (hertuzumab) conjugated with the microtubule inhibitor monomethyl auristatin E (MMAE) via a cleavable linker, has gained approval for the treatment of locally advanced or metastatic gastric cancer and urothelial cancer [36–38]. However, no ADCs have been approved for marketing so far for advanced cutaneous melanoma.

In this study, we aim to investigate the therapeutic efficacy and molecular mechanism underlying RC48 alone or in combination with dabrafenib in both in vitro and in vivo models of BRAF-mutant cutaneous melanoma. Our findings establish a preclinical foundation for the combined use of an anti-HER2 drug conjugate and a BRAF inhibitor for the treatment of BRAF-mutant cutaneous melanoma.

## Materials and methods

### Reagents and antibodies

RC48 was purchased from RemeGen Co. Ltd. (Shandong, China). Dabrafenib was purchased from Selleck (Houston, USA). Annexin V-FITC/PI cell apoptosis assay kit, Cell Cycle Assay Kit, BeyoClick™ EdU-555 cell proliferation assay kit, TUNEL assay kit, and crystal violet staining solution were purchased from Beyotime Biotechnology Inc (Shanghai, China).

### Cell lines

A2058 and SK-MEL-28 human melanoma cell lines were purchased from icell Bioscience Inc. (Shanghai, China). The identity of the cells was verified by short tandem repeats (STR) and the absence of mycoplasma contamination was confirmed. The cells were incubated in RPMI 1640/DMEM (Sigma, Germany) with 10% fetal bovine serum (FBS) (Gibco, USA) and 1% penicillin–streptomycin at 37 °C under 5% CO_2_.

### Cell viability assay

The A2058 and SK-MEL-28 cells were seeded in 96-well plates at a density of 2.0 × 10^3^ cells per well. After treatment with RC48 for 72 h, cell viability was determined using the Celltiter-Glo assay kit (Promega, USA) according to the protocol, and the Luminescence value (L) were measured using a multifunctional microplate reader (TECAN, Switzerland). The untreated cells served as a control. The cell survival rate (%) was calculated using the following formula: L_sample_/L_control_ × 100%. A nonlinear regression model was used to plot the s-shaped dose–survival curves and calculate IC_50_ values using a nonlinear regression analysis (GraphPad Prims 5.0).

The coefficient of drug interaction (CDI) was calculated to evaluate the interaction between two drugs, as reported previously [39]. The CDI was calculated relative to cell survival rate of each group as follows: CDI = AB/(A × B). AB is the cell survival rate of the combination group, and A and B are the cell survival rate of each single-agent group, respectively. CDI value < , = , or > 1 represents a synergistic, additive, or antagonistic effect, respectively.

### IncuCyte™ cell proliferation assay

A total of 2 × 10^3^ cells were seeded into 96-well plates, and then treated with the cells were added with RC48 or/and dabrafenib. Cell cultures were imaged every 2 h using IncuCyte ZOOM (Sartorius, Germany), which were housed inside a cell incubator at 37 ℃/5% CO_2_. Cell proliferation was evaluated as the percentage of cell density observed over this period. The results of the cell proliferation were analyzed 72 h later.

### Cell proliferation assay

A2058 and SK-MEL-28 cells were seeded in 12-well plates at a density of 5.0 × 10^4^ cells/well, and then incubated with/without indicated compounds for 24 h. Cell proliferation was estimated using an EdU Cell proliferation Kit with Alexa Flour 555 following the protocol. Images were captured using a laser scanning confocal microscope (Zeiss, Germany).

### Immunofluorescence

Cells were seeded onto the coverslips and fixed with 4% paraformaldehyde for 30 min. A 0.1% Tween-20 in phosphate-buffered saline (PBS) buffer was incubated at room temperature for 10 min. Blocking was performed using 5% bovine serum albumin in PBS buffer. Then, cells were incubated with indicated primary antibodies at 4 ℃ overnight. Alexa Flour 488-labeled secondary antibodies were incubated at room temperature for 1 h. Finally, the coverslips were sealed to protect from fluorescence quenching, and the images were captured using a fluorescence microscope (Leica, Germany).

### Cell apoptosis analysis

Cells were seeded at a density of 1 × 10^5^ cells/well in 12-well plates and incubated overnight, followed by treatment with indicated compounds for 48 h. The cells were collected and washed with PBS. Cell samples were stained using the Annexin V-FITC/PI cell apoptosis assay kit according to the protocol and analyzed using a FACSCanto II^TM^ cytometer (BD Biosciences, USA). Flowjo software was used to analyze the data.

### Cell cycle analysis

Cells were seeded at a density of 2 × 10^5^ cells/well in a six-well plate and treated with or without indicated compounds for up to 48 h. The cells were washed with cold PBS buffer and fixed with ice-cold 70% ethanol at 4 ℃ overnight. Cell samples were stained with propidium iodide according to the protocol for the Cell Cycle Assay Kit at 37 ℃ for 30 min. The proportions of cells in different phases of the cell cycle were analyzed using a FACSCanto II™ cytometer.

### Colony formation assay

A2058 and SK-MEL-28 cells were plated in six-well plates at a density of 5.0 × 10^3^ cells/well. The indicated compounds were then added with or without. After 14 days, the colonies were fixed by 4% paraformaldehyde for 30 min and stained with crystal violet for 10 min at room temperature. Images of the colonies were captured by a camera and the colony formation rate was calculated using ImageJ software.

### Cell migration and invasion assay

For cell migration assay, A2058 and SK-MEL-28 cells were plated at a density of 2.0 × 10^4^ in the upper chamber of a 24-well Transwell plate (Corning, USA) treated with RC48 and/or dabrafenib. Then, 500 μL DMEM or RPMI 1640 medium with 10% FBS was added to the lower chamber. After incubation for 24 h, 4% paraformaldehyde was added to the upper chamber to fix the cells for 30 min. Crystal violet was then used to stain for 10 min. For cell invasion assay, the upper chamber membranes were coated with Matrigel (Corning), and other experimental procedure was in line with the cell migration assay. The image were acquired by an inverted microscope (Leica, Germany). The cell migration and invasion rate were calculated using ImageJ software, respectively.

### Internalization

A2058 and SK-MEL-28 cells were inoculated in 12-well plates. RC48 (2 μg/mL) was incubated with the cells at 4 ℃ for 1 h, then the cells were incubated at 37 ℃ for 2 h and 24 h. The cells were incubated with 1:200 LAMP1 Polyclonal Antibody at 4℃ for 1 h. Finally, the cells were incubated with 1:200 goat anti-human IgG (H + L) cross-adsorbed secondary antibody labeled with Alexa Fluor 488 (green) (Invitrogen) and goat anti-rabbit IgG (H + L) highly cross-adsorbed secondary antibody labeled with Alexa Fluor 568 (red) at 4 °C for 1 h, the cells were fixed with 4% paraformaldehyde for 30 min and nuclei were counterstained with DAPI. The images were captured by using a confocal laser scanning microscope (Leica, Germany).

### Western blot

Cells and tissue proteins were lysed using sodium dodecyl sulfate (SDS) lysis buffer (Beyotime Biotechnology, Shanghai, China) supplemented with PMSF (Solarbio, Beijing, China) and Phosphatase Inhibitor Cocktail (Beyotime Biotechnology, Shanghai, China). Proteins were separated using SDS polyacrylamide gel electrophoresis (PAGE) and transferred to PVDF membranes (Epizyme, China). Next, the membranes were cut horizontally, incubated with primary and secondary antibodies. The protein bands were detected using ECL reagent (Cytiva, USA). Images were captured using a Bio-Rad Multifunctional chemiluminescence imaging system. GAPDH or β-tubulin was used as a loading control. Antibody information is shown in Additional file 1: Table S1.

### Hematoxylin and eosin (H&E) staining and immunohistochemistry

Normal tissue from the mice was fixed with 10% neutral formalin, dehydrated, and embedded in paraffin. After cutting into 4-µm thick sections, the essential organ slices were dewaxed using gradient alcohol and stained with hematoxylin and eosin. The histological structure was evaluated using a panoramic tissue quantitative analysis system (Zeiss, Tissue FAXS PLUS).

For immunohistochemistry (IHC) analysis, tumor tissues were fixed with 10% neutral formalin and embedded in paraffin. The anti-Ki67 antibody (1:1000) was incubated overnight at 4 ℃, followed by incubation with the horseradish peroxidase (HRP)-labeled secondary antibody for 1 h at room temperature. Finally, slides were stained with diaminobenzidine and counterstained with hematoxylin. The images were captured using a panoramic tissue quantitative analysis system.

### RNA sequencing analysis

For RNA sequencing (RNA-seq), A2058 cells were treated with RC48 and/or dabrafenib in six-well plates and RNA was isolated after 48 h of incubation. The RNA samples then underwent strict quality control, including accurate detection of RNA integrity using the Agilent 2100 bioanalyzer. mRNA was purified from total RNA using poly-Toligo magnetic beads, and library fragments were purified using the AMPure XP system. Complementary DNA (cDNA) fragments with a length of 370–420 bp were used. After constructing the library, initial quantification was performed using the Qubit 2.0 Fluorometer. The library was diluted to 1.5 ng/μL, and the insert size of the library was tested using the Agilent 2100 bioanalyzer. Once the insert size met expectations, quantitative reverse transcription polymerase chain reaction (qRT–PCR) accurately quantified the effective concentration of the library (ensuring it was higher than 1.5 nM) to ensure library quality. After passing the qualified library inspection, Illumina sequencing was performed by pooling different libraries according to the requirements of effective concentration and target data volume, generating 150 bp paired-end reads. The RNA-seq data is available at GEO (GSE252346).

### In vivo studies

A total of 180 old female BALB/C nude mice, aged 5–6 weeks, were purchased from SJA Laboratory Animal Co., Ltd, (Hunan, China). Mice were housed in individual ventilated cages (IVC) under specific pathogen-free (SPF) experimental conditions and provided ad libitum access to food and water in a room maintained at controlled temperature and humidity on a 12-h dark/light cycle. All animal experiments were performed in full compliance with the guidelines approved by the Biomedical Research Ethics Committee, Gannan Medical University on 16 October 2021 (no. 2021568).

To establish melanoma xenograft models, 3 × 10^6^ A2058 cells were suspended in 100 μL PBS and injected subcutaneously into 6–7 female BALB/C nude mice. When the mean volume of the tumors reached approximately 100–150 mm^3^, the mice were randomly divided into four groups (five mice per group), and injected intravenously with saline, 5 mg/kg of RC48, 10 mg/kg of RC48, and 10 mg/kg of trastuzumab once weekly (QW) three times in monotherapies. For the combination therapy study, the mice were administered with saline, 5 mg/kg RC48 QW three times, and dabrafenib, at a dose of 20 mg/kg, was injected intravenously five times every week for three weeks, and a combination of RC48 and dabrafenib. The tumor sizes and body weight of the mice were measured twice a week using a caliper until the tumor volume reached 1500 mm^3^, and the tumor volumes were calculated using the formula: tumor volume (mm^3^) = length × (width)^2^ × 0.5. The inhibition rate of the tumor growth (TGI) was calculated as (1-treated tumor volume/control tumor volume) × 100%. If the tumor volume did not exceed 1500 mm^3^ by the end of the experiment, the mice were euthanized, and organs were collected for subsequent analyses.

### Statistical analysis

All data were analyzed using GraphPad Prism 5. The mean ± standard error of the mean (SEM) was used to represent the results. A two-tailed Student’s *t*-test was utilized to determine significant p-values for comparisons between two groups. Differences were considered statistically significant when P-values were less than 0.05.

## Results

### RC48 significantly showed potent antiproliferative effects in HER2-positive melanoma cells in vitro

**Fig. 1:**
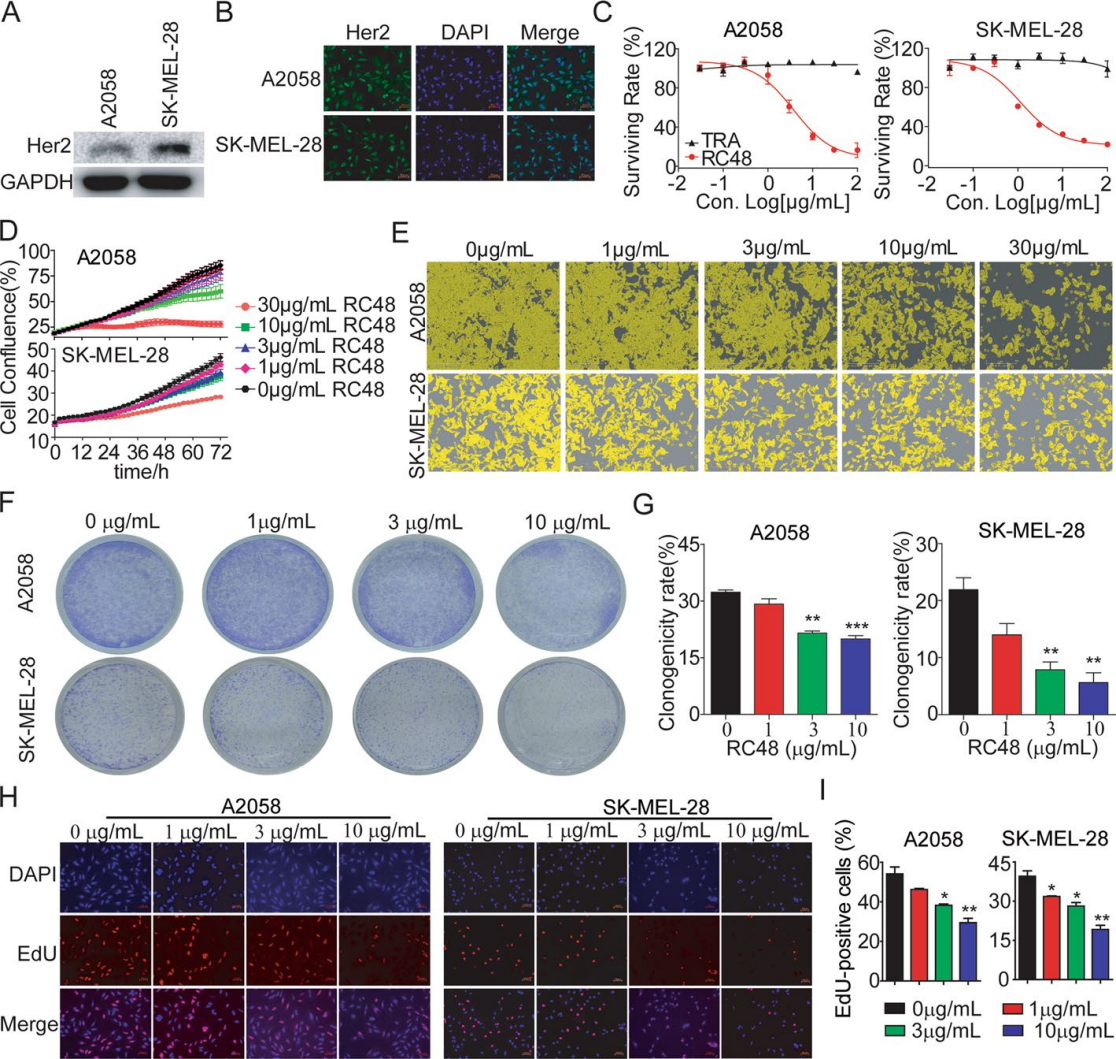
RC48 inhibited tumor cell growth in HER2-positive melanoma cells in vitro. **A** Quantification of HER2 protein expression in A2058 and SK-MEL-28 melanoma cell lines using western blotting. **B** The subcellular localization of HER2 protein (shown in green) in the A2058 and SK-MEL-28 cells. HER2 is localized to both the plasma membrane and the nucleus. It was determined using immunofluorescence. DAPI (shown in blue) was used to stain nuclei. The images were captured at 200× magnification, with scale bars set at 50 µm. **C** The A2058 and SK-MEL-28 cells were treated with RC48 and trastuzumab for 72 h. The cell viability was evaluated using the Cell Titer-Glo cytotoxicity assays. **D**, **E** The A2058 and SK-MEL-28 cells were treated with indicated concentrations of RC48. The cell confluency (%) was calculated from 0 to 72 h using Incucyte S3 Zoom software based on phase-contrast images. **F**–**G** The A2058 and SK-MEL-28 cells were treated with RC48 at concentrations of 0, 1, 3 and 10 μg/mL for 14 days. The area (%) of colonies stained with crystal violet was used to measure the antiproliferative effects. **H**, **I** The A2058 and SK-MEL-28 cells were incubated with RC48 at concentrations of 0, 1, 3 and 10 μg/mL for 24 h. Then, the cells were stained with Azide 555 (shown in red) to detect EdU and DAPI (shown in blue). Fluorescence images were obtained and analyzed using a confocal laser scanning microscope at 200× magnification, with scale bars set at 50 µm. The data presented are the means ± SEM from three independent experiments, and statistical significance was determined using an unpaired *t*-test (*p < 0.05; **p < 0.01; ***p < 0.001 compared with control groups)

To screen for relevant melanoma cell lines for in vitro and in vivo evaluation, we firstly validated protein expression of HER2 in A2058 and SK-MEL-28 melanoma cell lines using western blot and immunofluorescence analysis. Our results showed that HER2 was overexpressed and its subcellular localization was confirmed in A2058 and SK-MEL-28 cell lines. We found that HER2 was localized on the cell membrane or cytoplasm (Fig. 1A and B). Internalization is a crucial step for the efficacy of most ADC drugs, especially HER2-targeting ADCs such as T-DM1 [40, 41]. In the case of RC48, it is also well established that RC48 undergoes target-mediated endocytosis once it binds to HER2 on the surface of breast cancer cells [36]. In support of this, we performed endocytosis analysis and showed that RC48 can be internalized upon HER2 binding in A2058 and SK-MEL-28 cells (Additional file 1: Fig. S1). To investigate whether RC48 effectively inhibits the viability of HER2-positive melanoma cells, A2058 and SK-MEL-28 cells were exposed to different concentrations of RC48 and trastuzumab for 72 h. RC48 demonstrated significant antiproliferative activity in a dose-dependent manner by Cell Titer-Glo cytotoxicity assays. The IC_50_ values for A2058 and SK-MEL-28 melanoma cells were 7.35 ± 3.62 μg/mL [maximum-dose inhibitory rate (MIR) of 87.44 ± 3.74%] and 1.23 ± 0.08 μg/mL (MIR of 77.06 ± 1.24%), respectively. In contrast, trastuzumab showed no apparent cytotoxicity in these HER2-positive melanoma cell lines (Fig. 1C). Further examination of dose- and time-dependent effects of RC48 and trastuzumab on these cells was conducted using the cell confluence using IncuCyte Live Cell Imaging system. Similar dose responses were observed in A2058 and SK-MEL-28 cells (Fig. 1D and E, Additional file 1: Fig. S2).

To further evaluate the antiproliferative effect of RC48, A2058 and SK-MEL-28 cells were treated with RC48 via clonogenic assay and EdU staining assay. Our data revealed that RC48 significantly inhibited the proliferation of A2058 and SK-MEL-28 melanoma cells in dose-dependent manner (Fig. 1F–I). Therefore, these results indicated that RC48 exhibited potent antitumor activity in melanoma cells in vitro.

### RC48 prominently induced apoptosis, cell-cycle arrest, and cell motility in melanoma cells

**Fig. 2:**
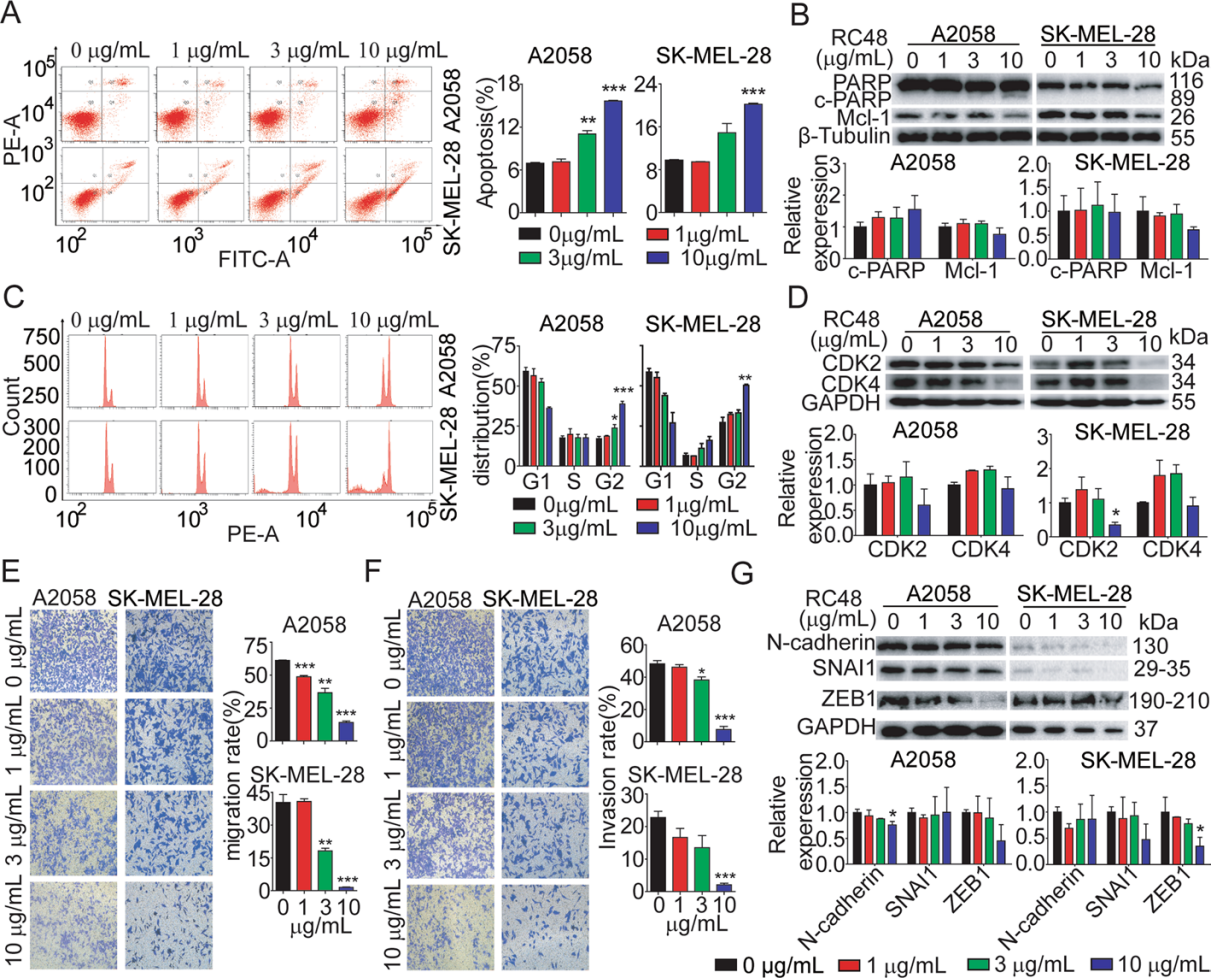
RC48 induced cell cycle arrest, apoptosis and inhibited cell motility in melanoma cells. **A** The A2058 and SK-MEL-28 cells were treated with RC48 at concentrations of 0, 1, 3, and 10 μg/mL for 48 h. The cells were stained with an anti-Annexin V-FITC antibody and PI for apoptosis analysis using flow cytometry. The percentage of Annexin V^+^ PI^−^ and Annexin V^+^ PI^+^ cells (apoptosis cells) is shown in right bar chart. **B** The protein levels of apoptosis markers, including poly ADP-ribose polymerase (PARP), cleaved PARP, and Mcl-1 were detected using western blot analysis. β-tubulin was used as a loading control. **C** A2058 and SK-MEL-28 cells were treated with RC48 at concentrations of 0, 1, 3, and 10 μg/mL. The induction of cell cycle changes was analyzed using flow cytometry. The percentage of cells in each phase of the cell cycle was shown in a bar chart. **D** The protein levels of cell cycle regulators, CDK2 and CDK4, were detected using western blot. GAPDH was used as a loading control. **E**, **F** A2058 and SK-MEL-28 cells were treated with RC48 at concentrations of 0, 1, 3, and 10 μg/mL for 24 h. Cell motility was assessed using Transwell migration (**E**) and invasion (**F**) assays. **G** A2058 and SK-MEL-28 cells were treated with different concentrations of RC48 for 24 h. The expression of epithelial–mesenchymal transition (EMT) markers, including N-cadherin, SNAI1, and ZEB1, was examined using western blot analysis. GAPDH was used as a loading control. The data presented represent the mean ± SEM of at least three independent experiments. The statistical significance was assessed using an unpaired *t*-test (*p < 0.05, **p < 0.01, and ***p < 0.001 compared with control groups)

To investigate the underlying mechanism of antitumor action of RC48 in vitro, we evaluated the impact of RC48 on apoptosis, cell cycle, and cell motility in A2058 and SK-MEL-28 cells. Apoptosis of melanoma cells was obvious after 0, 1, 3 and 10 g/mL RC48 treatment for 48 h, and the proportion of Annexin V^+^ cells increased in a dose-dependent manner (Fig. 2A). At the protein level, dose-dependent increased expression of cleaved-PARP and decreased expression of apoptosis-inhibiting proteins Mcl-1, compared with the control group, were observed in A2058 and SK-MEL-28 cells (Fig. 2B). Furthermore, after treatment with 0, 1, 3, and 10 g/mL RC48 for 48 h, RC48 significantly induced cell cycle changes in a dose-dependent manner characterized by a decrease in G0–G1 phase and a concomitant increase in G2–M phase in A2058 and SK-MEL-28 cells, indicative of cell cycle arrest in the G2–M phase (Fig. 2C). A dose-dependent cell cycle arrest in these cells treated with RC48 was evidenced by decreased expression of the CDK2 and CDK4 (Fig. 2D).

Tumor cell dissemination is one of the basic characteristics of malignant tumors, and it the main reason for tumor recurrence and distant metastasis, thus seriously influencing the prognosis and survival of cancer patients [42, 43]. Next, we examined the effect of RC48 on cell motility in vitro. A2058 and SK-MEL-28 cells were treated with RC48 using Transwell assays. As observed, RC48 dose-dependently inhibited the migration and invasion abilities in A2058 and SK-MEL-28 cells compared with the untreated group (Fig. 2E, F). Epithelial to mesenchymal transition (EMT) is an important pathway for invasion and migration of epithelial cell tumors. EMT markers such as N-cadherin, SNAI1, and ZEB1 were decreased in the tested cells (Fig. 2G).

Taken together, these findings displayed that RC48 inhibited tumor cell proliferation by inducing cell cycle and apoptosis, and inhibiting migration and invasion, ultimately leading to melanoma cell death.

### RC48 significantly inhibited the growth of melanoma in vivo

**Fig. 3:**
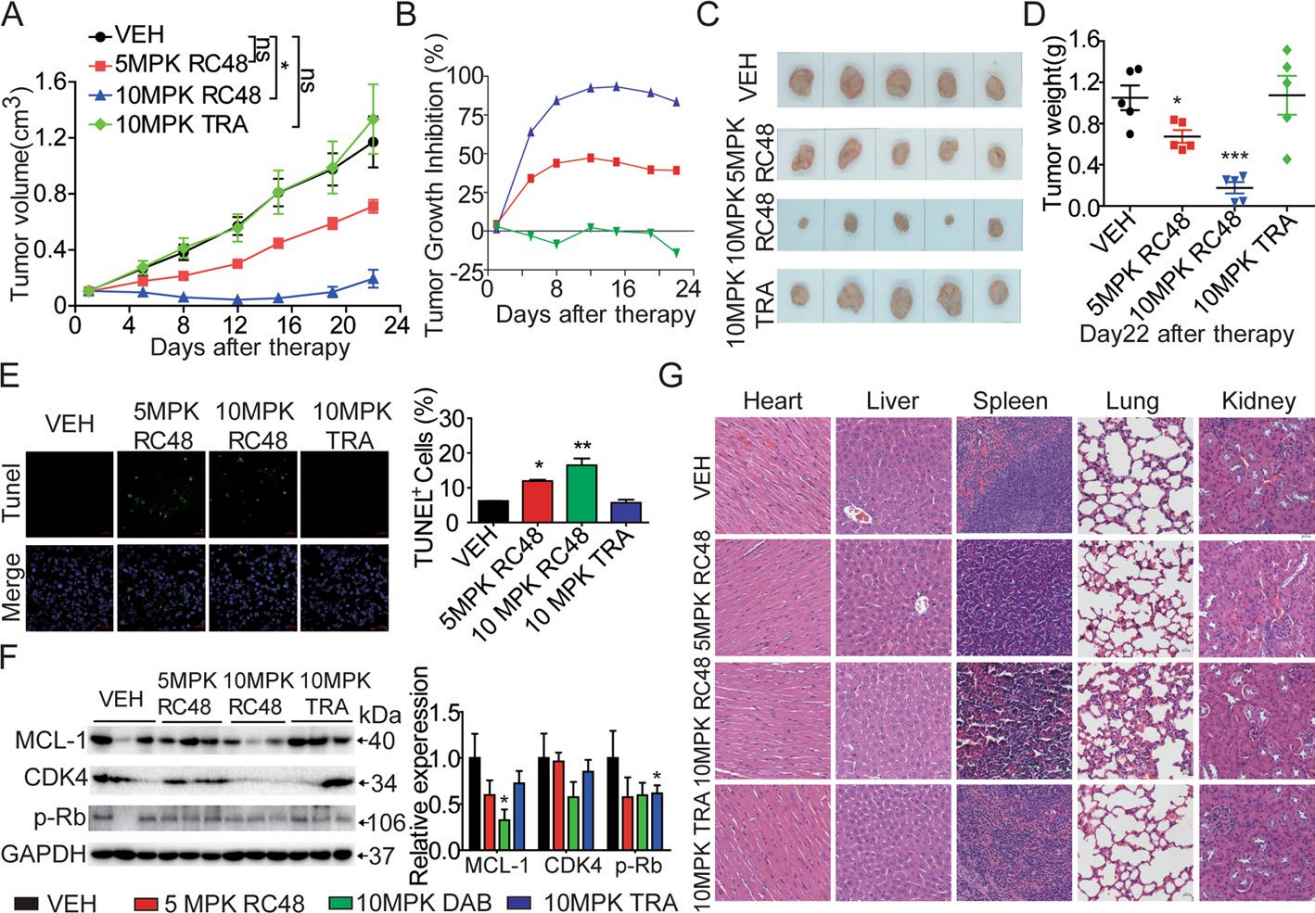
RC48 remarkably exerted antitumor activity in the A2058 CDX model. BALB/c nude mice were injected subcutaneously with 2 × 10^6^ A2058 cells. Mice with xenografts measuring approximately 100–150 mm^3^ were randomly allocated into four groups, including vehicle (VEH), RC48 (5 MPK and 10 MPK) and 10 mg/kg trastuzumab (10 MPK TRA). Tumor sizes and body weight were measured twice a week. **A** Tumor growth in the different treatment groups was evaluated in A2058 CDX model by caliper measurements. **B**–**D** Tumor growth inhibition (%), representative transplanted tumor weights and images were assessed at the end of the experiment. **E** The TUNEL assay showed a higher rate of cell apoptosis in the RC48 group compared with the vehicle and trastuzumab groups. TUNEL images were captured at 400× magnification, with a scale bar of 20 µm. **F** The protein levels of Mcl-1, CDK4, and p-Rb in xenograft tumor tissue were examined using immunoblot analysis. GAPDH was used as a loading control. **G** Pathological changes in these organs of A2058 mouse CDX model were evaluated using H&E staining assays. Images were captured at 400× magnification. Scale bars, 20 µm. Data represents the mean ± SEM of at least three independent experiments, and statistical significance was assessed using an unpaired *t*-test, *p < 0.05; **p < 0.01; ***p < 0.001 compared with control groups

To determine the tumoricidal activity of RC48, we conducted an in vivo study using mouse xenograft models of human melanoma. We established BALB/c nude mouse subcutaneous xenografts of A2058 melanoma cells and allowed the tumors to develop until their average volumes reached at least 100 mm^3^. The tumor-bearing mice were then randomly divided into four groups. Intravenous administration via the tail vein was performed with the following substances: vehicle (VEH, administered weekly for 3 weeks), 10 mg/kg trastuzumab (administered weekly for 3 weeks 10 MPK TRA), and RC48 (administered at doses of 5 MPK and 10 MPK, weekly for three weeks). In the A2058 CDX model, RC48 significantly induced tumor regression. Terminal tumor growth inhibition (TGI) of 39.25% and 83.40% was observed at doses of 5 mg/kg and 10 mg/kg RC48, respectively. Tumors in the vehicle group grew rapidly, reaching a volume of 1170 ± 182 mm^3^ and weight of 1.05 ± 0.12 g by day 22. On the other hand, RC48 demonstrated dose-dependent and substantial antitumor activity. In the 5 mg/kg RC48 group, the mean tumor size reached 711 ± 46 mm^3^ (*p* = 0.0404) and a weight of 0.67 ± 0.06 g (*p* = 0.024). In the 10 mg/kg RC48 group, the mean tumor size reached 194 ± 64 mm^3^ (*p* = 0.00098) and a weight of 0.18 ± 0.06 g (*p* = 0.00017). However, the administration of 10 mg/kg trastuzumab did not show any inhibitory effect on tumor growth and resulted in a tumor size of 1334 ± 249 mm^3^ and a weight of 1.07 ± 0.19 g (Fig. 3A–D).

All the A2058 xenograft tumors were collected and prepared for TUNEL, western blot, and H&E analysis. Immunofluorescence images displayed a strong TUNEL signal in the 5 and 10 mg/kg RC48 groups, but not in the vehicle and trastuzumab groups (Fig. 3E). Western blot analysis showed a significant decrease in the expression levels of MCL-1, CDK4, and phosphorylated Rb in A2058 xenograft tumor of the 5 and 10 mg/kg RC48 groups compared with the vehicle groups (Fig. 3F). In addition, histopathological images of the dissected organs of the mice showed that RC48 did not have any notable toxic side-effects on the heart, liver, spleen, lung, and kidney (Fig. 3G).

Therefore, these results from the in vivo experiments are consistent with the in vitro findings and support the potential antitumor activity of RC48 alone.

### RC48 synergized with dabrafenib to inhibit melanoma cell growth in vitro

It is worth mentioning that the combination therapy of ADC and other anticancer drugs has become a significant focus in the development of ADC drugs [44]. Based on this, we hypothesize that RC48, either alone or in combination with dabrafenib, a clinical stage BRAF inhibitor, may offer improved clinical benefits for patients with BRAF-mutant cutaneous melanoma.

**Fig. 4:**
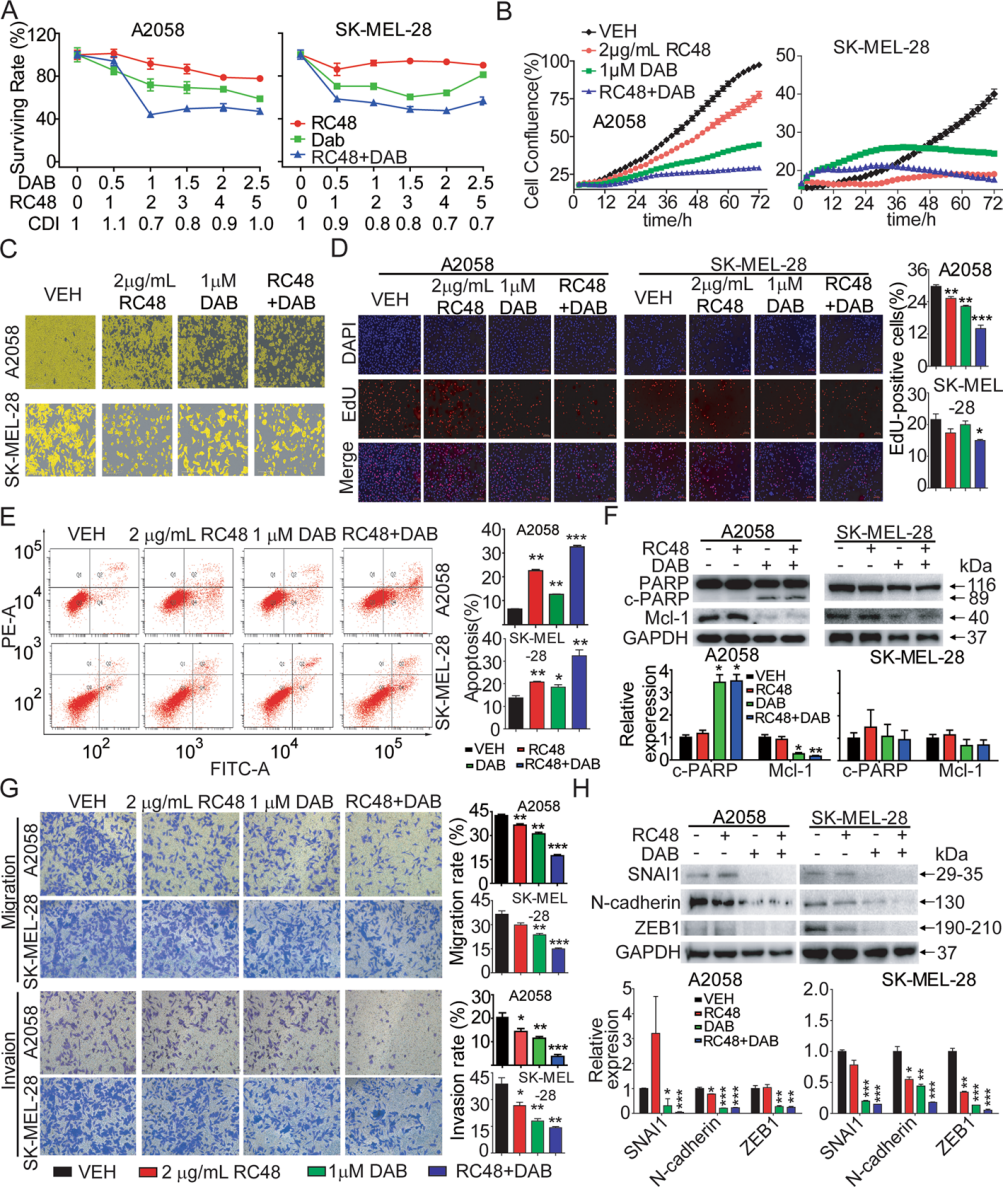
RC48 and dabrafenib synergistically inhibited the antitumor activity of A2058 and SK-MEL-28 cells in vitro. **A** Cytotoxicity of a combination with RC48 and dabrafenib (DAB) in A2058 and SK-MEL-28 cells as determined by CellTiter-Glo® Assay in accordance with the manufacturer’s instructions. CDI < 1 represents a synergistic effect. **B** A2058 and SK-MEL-28 cells were incubated with 2 μg/mL RC48, 1 μM DAB, or RC48 + DAB for various times. The cell confluency (%) was calculated using Incucyte S3 Zoom software based on phase contrast images from 0 h to 72 h. **C** The cell confluency (%) was shown for RC48 and DAB alone or in combination at 72 h. **D** A2058 and SK-MEL-28 cells were incubated with 2 μg/mL RC48, 1 μM DAB, or their combination for 24 h. Then A2058 cells were stained with Azide 555 (red) to detect EdU and DAPI (blue). Fluorescence images were obtained and analyzed with a confocal laser scanning microscope. Images captured at 200× magnification. Scale bars, 50 µm. **E** A2058 and SK-MEL-28 cells were treated with different treatments for 48 h. The cells were stained with an anti-Annexin V-FITC antibody and PI for apoptosis analysis using flow cytometry. **F** A2058 and SK-MEL-28 cells were treated with different treatments for 48 h. Western blotting was used to examine the expression levels of PARP and Mcl-1. GAPDH was used as a loading control. **G** A2058 and SK-MEL-28 cells were exposed to different treatments for 24 h. Cell motility was detected using Transwell migration and invasion assays. **H** A2058 and SK-MEL-28 cells were exposed to different treatments of 2 μg/mL RC48, 1 μM DAB, or RC48 + DAB for 24 h. Western blotting was used to examine the expression of EMT markers (SNAI1, N-cadherin, ZEB1). GAPDH was used as a loading control. Data represent the mean ± SEM of at least three independent experiments, and statistical significance was assessed using an unpaired *t*-test; *p < 0.05, **p < 0.01, ***p < 0.001 compared with control groups

To test this hypothesis, we treated A2058 and SK-MEL-28 human cutaneous melanoma cells, which harbor the BRAF (V600E) mutation [45–48], with RC48 alone or in combination with dabrafenib. RC48 alone was tested at concentrations of 0, 1, 2, 3, 4, and 5 μg/mL, while dabrafenib alone was tested at concentrations of 0, 0.5, 1, 1.5, 2, and 2.5 μM, as determined by Cell Titer-Glo cytotoxicity assays. Several combination doses showed synergistic effects in A2058 and SK-MEL-28 cells. Among them, the combination treatment of 2 μg/mL RC48 and 1 μM dabrafenib showed the most synergetic effects in these cells (Fig. 4A). Therefore, we selected 2 μg/mL RC48 alone or in combination with 1 μM dabrafenib for subsequent in vitro experiments. Further analysis using the IncuCyte Live Cell Imaging system showed that RC48 plus dabrafenib synergistically resulted in growth-inhibitory potency compared with RC48 or dabrafenib monotherapy (Fig. 4B, C). Additionally, EdU staining revealed that RC48 combined with dabrafenib significantly inhibited the proliferation of A2058 and SK-MEL-28 cells compared with all other monotherapy groups (Fig. 4D).

To further explore whether the synergistic growth inhibition was induced by combination of RC48 and dabrafenib in vitro, we evaluated the effect of combined therapy on apoptosis, cell cycle and cell motility in A2058 and SK-MEL-28 cells. Flow cytometry assays demonstrated that the combination of RC48 and dabrafenib cooperatively induced a significant increase in the percentage of apoptotic cells compared to treatment with 2 μg/mL RC48 or 1 μM dabrafenib alone (Fig. 4E). At the protein level, the combination of RC48 and dabrafenib resulted in increased expression of cleaved-PARP and decreased expression of the apoptosis-inhibiting protein Mcl-1 compared with all other treatment groups in both A2058 and SK-MEL-28 cells (Fig. 4F). Furthermore, Transwell cell migration/invasion assays showed that the combination treatment had a more pronounced inhibitory effect on the migration and invasion of melanomas cells compared to all other monotherapy groups (Fig. 4G). We also observed that the combination of RC48 with dabrafenib significantly decreased the protein levels of SNAI1, N-cadherin, and ZEB1, when compared with the single-agent treatment groups (Fig. 4H).

These results provided evidence that the synergistic inhibition effects observed with the combined therapy of RC48 and dabrafenib can be partially attributed to the induction of PARP and MCL-1-dependent apoptosis, and inhibition of migration and invasion in BRAF-mutant melanoma cells in vitro.

### Combination effects of RC48 and dabrafenib on global gene expression profiling

**Fig. 5:**
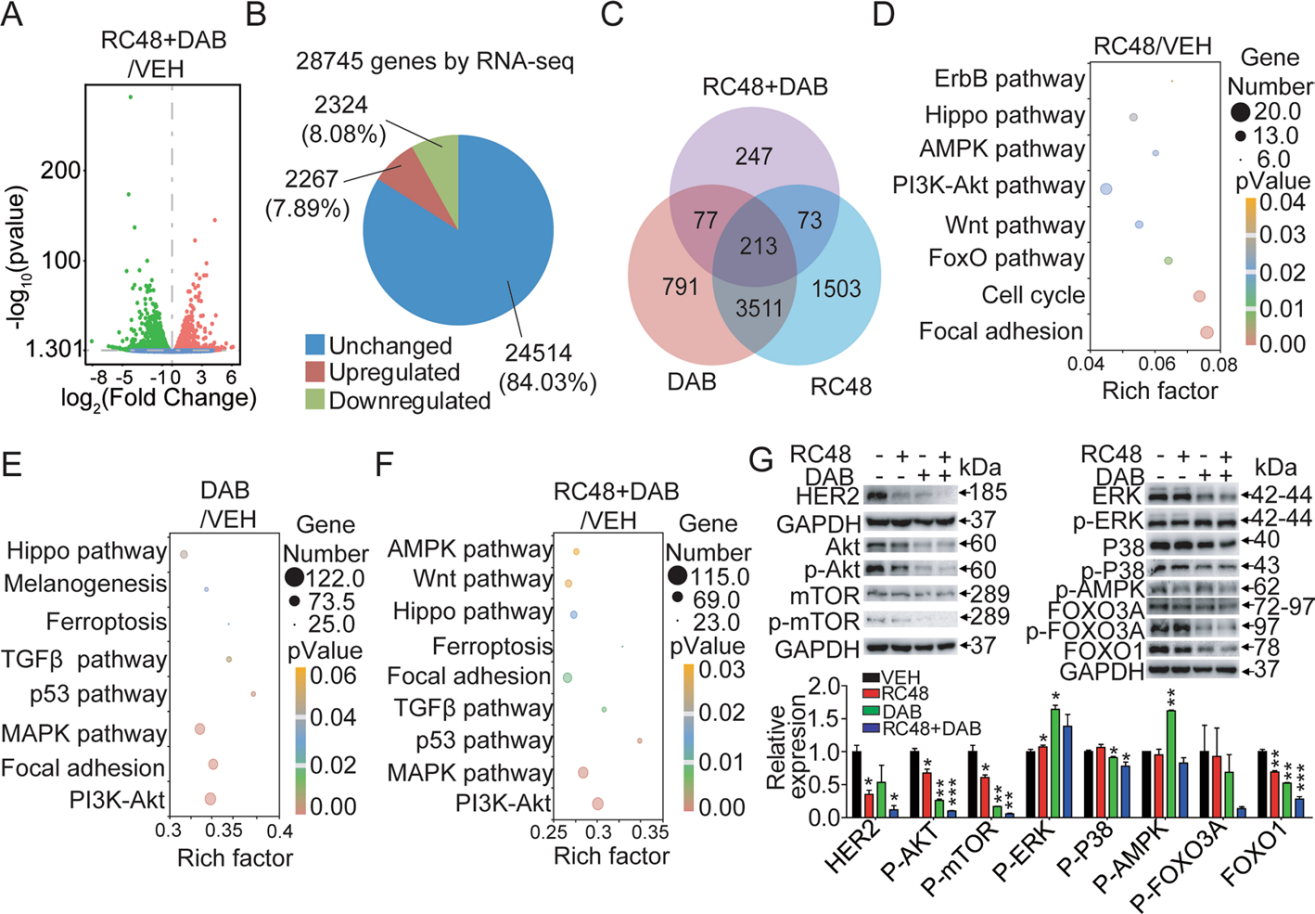
Effects of RC48 and dabrafenib combination treatment on global gene expression profile. Gene expression studies were conducted using RNA-seq in A2058 cells treated with vehicle control, 2 μg/mL RC48, 1 μM DAB, or their combination for 24 h. Each group represents triplicate samples. **A**, **B** The expression of DEGs in A2058 cells between the vehicle and RC48 plus DAB combination treatment. Upregulated and downregulated genes are shown in red and green, respectively. Values are presented as the log_10_ of tag counts. **C** Number of overlapping DEGs in cells treated with the combination compared to the VEH-treated cells. **D**–**F** Kyoto Encyclopedia of Genes and Genomes (KEGG) analysis of DEGs in the RC48 (**D**), DAB (**E**), and RC48 + DAB (**F**) treated samples compared with the VEH-treated samples in A2058 cells. **G** Analysis of the PI3K/AKT pathway, MAPK pathway, AMPK pathway, and FOXO pathway. GAPDH was used as the loading control. Statistical significance was assessed using an unpaired *t*-test; *p < 0.05, **p < 0.01, ***p < 0.001 compared with VEH groups

To further investigate the potential mechanisms underlying the synergistic effects, we performed bulk RNA-seq analysis in A2058 cells treated with 2 μg/mL RC48 and 1 μM dabrafenib, either alone or in combination, for 48 h. We analyzed the differentially expressed genes (DEGs) in each group using multidimensional scaling (Fig. 5A–C). In the combination treatment group, a significant number of genes exhibited differential expression compared to the vehicle group. Specifically, 4591 genes (15.97%) were found to be significantly differentially expressed in A2058 cells upon combinational treatment. Out of these genes, transcripts of 2267 genes (7.89%) were upregulated, whereas transcripts of 2324 (8.08%) were downregulated compared with the vehicle group.

Pathway analysis of the DEGs using KEGG revealed that RC48 treatment regulated multiple pathways in A2058 cells, including the Hippo pathway, PI3K-AKTpathway, AMPK pathway, Foxo pathway, and focal adhesion, among others. On the other hand, dabrafenib primarily modulated the Hippo pathway, p53 signaling pathway, MAPK pathway, PI3K-AKT pathway, and AMPK pathway, which were among the most significantly regulated genes. Importantly, the combination treatment also affected these key pathways, including the p53 signaling pathway, MAPK pathway, Hippo pathway, PI3K-AKT pathway, AMPK pathway, and focal adhesion, all of which played important roles in the in vitro antitumor effect (Fig. 5D–F, and Additional file 1: Fig. S3).

We further validated the combined effects of RC48 and dabrafenib on biological processes and related signaling pathways mentioned above in A2058 cells. Consistent with the RNA-seq analysis, when compared with RC48 or dabrafenib alone, the combined treatment of RC48 and dabrafenib decreased the phosphorylation of AKT, mTOR, ERK, P38, AMPK, FOXO3A, and FOXO1, indicating that RC48 plus dabrafenib inhibited all these pathways in A2058 cells.These changes in protein expression, related to apoptosis, also resulted in the inhibition of proliferation in A2058 cells (Fig. 5G).

Collectively, these data clearly demonstrated that RC48 plus dabrafenib inhibited multiple signaling pathways, such as p53 signaling pathway, MAPK pathway, PI3K-AKT pathway, AMPK pathway, etc., to exert its antitumor activity in A2058 cells.

### RC48 combined with dabrafenib treatment affected additional pathways and downregulated unique melanoma-associated prognostic genes

**Fig. 6:**
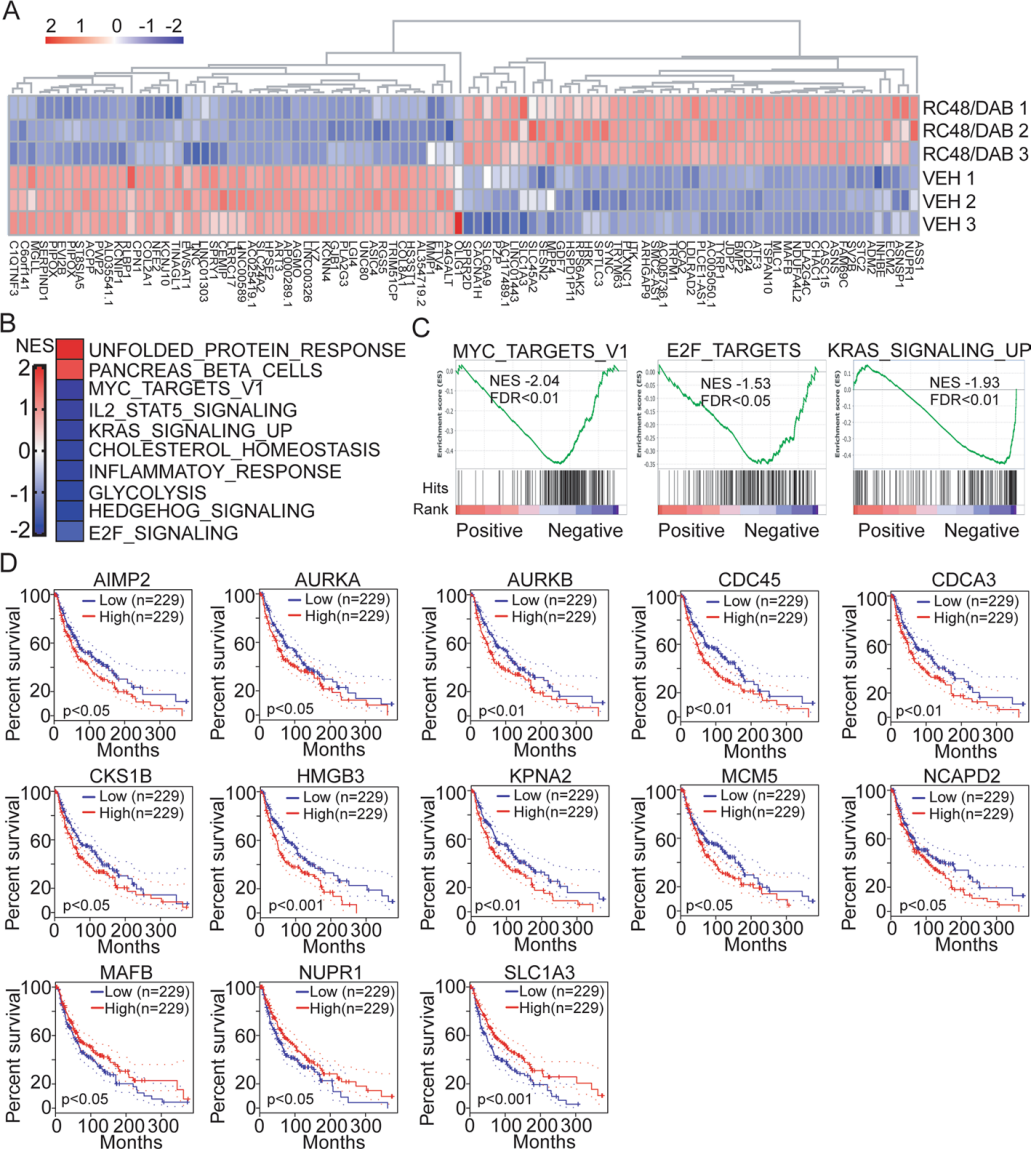
RC48 plus dabrafenib treatment affected additional pathways and downregulated unique melanoma-associated prognostic genes. **A** Heatmap showing the mRNA levels of the top 100 genes (50 downregulated or downregulated) after RC48 plus dabrafenib combination treatment. The expression levels of each gene were normalized to the total mRNA abundance of each sample and compared with that of vehicle-treated controls. **B** The top-ranked positively and negatively enriched gene sets identified using GSEA in response to combination treatment. GSEA was conducted with top common DEGs in A2058 cells after combinational treatment using 38 HALLMARK gene sets database in MSigDB. **C** GSEA plots showing strong negative enrichment of the MYC Targets v1, E2F_targets, and KRAS_signaling_up in A2058 cells in response to combination treatment. NES, normalized enrichment score; FDR, false discovery rate. **D** Kaplan–Meier estimate of overall survival based on expression of DEGs (TCGA melanoma Cohort). High relative expression of *AIMP2*, *AURKA*, *AURKB*, *CDC45*, *CDCA3*, *CKS1B*, *HMGB3*, *KPNA2*, *MCM5*, and *NCAPD2* genes, and low relative expression of *MAFB*, *NUPR1*, and *SLC1A3* were associated with poor overall survival in the melanoma cohort. The log rank (Mantel–Cox) test was used for significance, with *p* < 0.05 considered significant

Next, gene set enrichment analysis (GSEA) was conducted using the 38 Hallmark gene set collections in MSigDB to identify specifically enriched biological pathways after the combination treatment of RC48 and dabrafenib. The GSEA analysis of the common DEGs revealed strong negative enrichment in gene sets involved in MYC Targets v1, E2F_targets, and KRAS_signaling_up in A2058 cells treated with combination therapy (Fig. 6A–C). The upregulated and downregulated gene sets are presented in Fig. 6B and Additional file 1: Table S2.

To validate the biological relevance of the genes regulated by RC48 plus dabrafenib in melanoma, the TCGA dataset from the melanoma cohort was explored. This was done to confirm if any of the top 200 (100 up and down) DEGs identified by RNA-seq analysis were aberrantly expressed and/or associated with the outcome. Ten genes, including *AIMP2*, *AURKA*, *AURKB*, *CDC45*, *CDCA3*, *CKS1B*, *HMGB3*, *KPNA2*, *MCM5*, and *NCAPD2*, which were downregulated following combination therapy, were found to be overexpressed in the melanoma TCGA cohort. On the other hand, three genes (*MAFB*, *NUPR1*, and *SLC1A3*), which were upregulated following combination therapy, showed low expression in the melanoma TCGA cohort (Additional file 1: Fig. S4). Kaplan–Meier survival analysis indicated that the high or low expression of each of these genes was associated with poor overall survival in the melanoma cohort (*p* < 0.05) (Fig. 6D). Intriguingly, these genes that were downregulated in response to the RC48 and dabrafenib treatment, and were also observed to be overexpressed and associated with poor prognosis in melanoma TCGA cohort, were also components of the most enriched pathways identified by GSEA (E2F and MYC).

### In vivo combination therapy with RC48 and dabrafenib

**Fig. 7:**
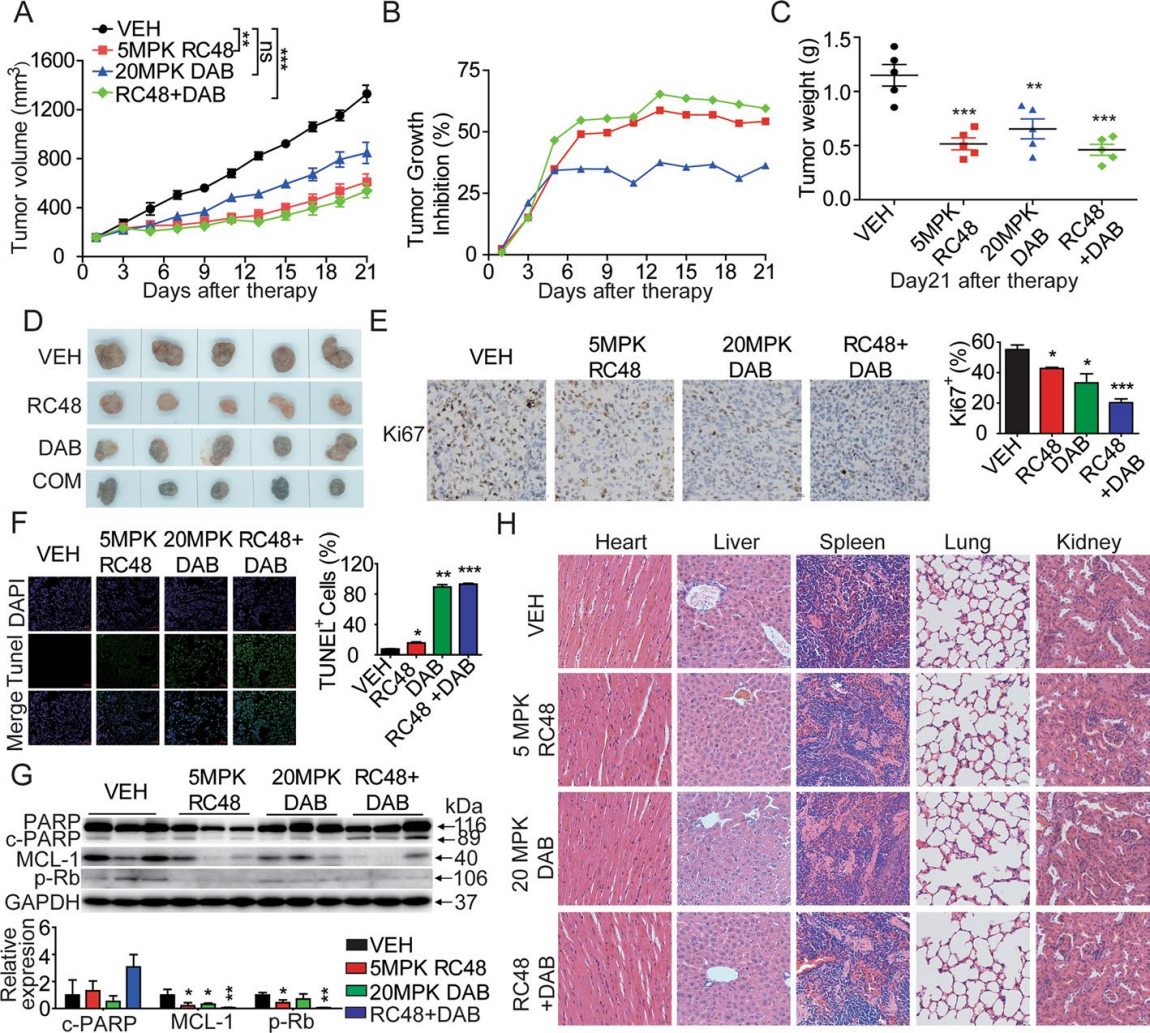
RC48 combined with dabrafenib potently inhibit tumor growth in A2058 CDX model in vivo. The A2058 CDX model was established in BALB/c-nude mice, and mice bearing xenografts averaging approximately 100–150 mm^3^ were randomly allocated into four groups: vehicle (VEH), 5 mg/kg RC48, 20 mg/kg DAB, and their combination. **A**, **B** Tumor growth curve and inhibition rate of tumor growth (TGI) in the A2058 CDX model. **C**, **D** A2058 transplanted tumor weights and tumor images were evaluated at the end of the experiment. **E** Images of immunohistochemical staining for Ki67 in A2058 xenograft tumors, IHC images captured at 400× magnification. Scale bars , 20 µm. **F** Immunofluorescence TUNEL images of of A2058 xenograft tumors captured at 200× magnification. Scale bars, 50 µm. **G** The expression levels of PARP, Mcl-1, and p-Rb in A2058 xenograft tumors were examined using immunoblot analysis. **H** Pathological changes in these organs of the A2058 mouse CDX model were evaluated using H&E staining assays. Images captured at 400× magnification. Scale bars , 20 µm. Data represent the mean ± SEM of at least three independent experiments, and statistical significance was assessed using an unpaired *t*-test (*p < 0.05; **p < 0.01; ***p < 0.001)

To further explore whether the in vitro combination therapeutic effects can be translated into an in vivo setting, we conducted further investigation on the antitumor activity of RC48 combined with dabrafenib in BALB/c nude mice bearing subcutaneous A2058 xenograft models. Our findings showed that the combination of 5 mg/kg RC48 and 20 mg/kg dabrafenib resulted in significantly higher tumoricidal activity in vivo, with a TGI of 59.55% observed on day 21. The mean tumor size in the combination group reached 538 ± 58 mm^3^ (*p* = 0.000024) and 1.05 ± 0.12 g (*p* = 0.00026). In contrast, tumors in the vehicle group grew rapidly and reached 1330 ± 70 mm^3^ and 1.05 ± 0.12 g. The mean tumor size in the 5 mg/kg RC48 group reached 608 ± 66 mm^3^ (*p* = 0.000069) and 0.67 ± 0.06 g (*p* = 0.00051), while in the 20 mg/kg dabrafenib group, it reached 847 ± 86 mm^3^ (*p* = 0.0025) and 0.18 ± 0.06 g (*p* = 0.0062) (Fig. 7A–D).

Furthermore, all the A2058 xenograft tumors were collected and prepared for IHC, TUNEL, western blot, and H&E assays. IHC staining showed a decreased expression level of Ki67 in the xenograft tumor tissues in the combination group of RC48 and dabrafenib compared with the monotherapy groups, indicating the antiproliferative activity of the combination treatment (Fig. 7E). Additionally, immunofluorescence images displayed a more intense TUNEL signal in the combined group (Fig. 7F). Moreover, these findings demonstrated a markedly decreased expression level of MCL-1 and phosphorylated Rb, and an increased expression level of cleaved-PARP in A2058 xenograft tumors treated with the combination of RC48 and dabrafenib compared to the single-agent treatment groups (Fig. 7G). Notably, histopathological images of the mouse organ dissections showed that the combination of RC48 and dabrafenib did not have notable toxic side-effects on the heart, liver, spleen, lung, and kidney (Fig. 7H). In agreement with the findings from the in vitro experiments, the results from the in vivo experiments provide support for the promising and superior antitumor effect of the combination of RC48 and dabrafenib.

Altogether, these aforementioned in vivo findings demonstrated that the combination therapy of RC48 and dabrafenib significantly inhibits melanoma growth in vivo, providing initial rationale for future clinical evaluations of the anti-HER2 drug conjugate, either alone or in combination with BRAF inhibitor, for the treatment of BRAF mutant cutaneous melanoma.

## Discussion

In our study, we showed that HER2 was expressed as a biomarker in response to RC48 in human cutaneous melanoma cells. Despite melanoma cells typically not overexpressing HER2, we confirmed the generally low expression of HER2 in human melanoma cells, consistent with previous reports in cell lines and clinical specimens of malignant melanoma [49, 50]. Despite this low expression, we showed that RC48 exhibited superior therapeutic efficacy by inducing cell cycle arrest, apoptosis, and inhibiting of cell motility in HER2-positive and BRAF-mutant cutaneous melanoma cells. Importantly, the in vivo experiments provided supporting evidence for the potential antitumor activity of RC48. Our findings are consistent with previous studies that have demonstrated the tumoricidal effects of RC48 on gastric cancer and breast carcinoma cell lines, xenograft models, and patient-derived xenograft (PDX) models with HER2 overexpression, even in cases with moderate to low expression of HER2 [21–23].

HER2 has been considered to be one of the most successfully targeted therapeutic markers expressed in various tumor tissues [23, 51, 52]. It is important to note that HER2 expression in melanoma is generally low [53]. Unlike in breast cancer, where HER2 overexpression is well-established and successfully targeted by therapies like trastuzumab, only rare individual cases of melanoma exhibit HER2 alterations that lead to HER2 overexpression. This low expression of HER2 in melanoma could explain the lack of efficacy observed with HER2-targeted therapies such as trastuzumab [53]. Of note, ADCs can bind to the tumor associated antigen on the cell surface and are subsequently endocytosed into lysosomes to release the toxin payload by cleaving the protease-mediated linker to inhibit tumor cells. Although the therapeutic efficacy of HER2-targeting ADCs (such as T-Dxd) is HER2-dependent, high expression of HER2 is not necessary. Modi et al. provided further evidence in the DESTINY-Breast04 Clinical Trials (gov. number, NCT03734029) that T-DXd resulted in significantly longer progression-free and overall survival for patients with HER2-low metastatic breast cancer [54]. In line with this understanding, our results suggested that RC48, either alone or in combination with dabrafenib, has the potential to improve treatment outcomes for HER2-low patients with BRAF-mutated cutaneous melanoma.

Combination therapy, in which certain drugs with different mechanisms are coadministered, is widely used in biomedical research and clinical applications [55]. Combination therapy involving BRAF and MEK inhibition has shown improved survival outcomes compared with using the BRAF inhibitor alone [56]. ADCs have emerged as a new class of anticancer drugs, approved for the treatment of solid and hematological malignancies as single agents. However, there is an increasing interest in combining ADCs with other anticancer drugs to enhance treatment effectiveness [44, 57]. In this study, we evaluated the coadministration of RC48 and dabrafenib for treating BRAF-mutant melanoma. We showed a synergistic antitumor effect of RC48 and dabrafenib in both in vitro and in vivo models of BRAF-mutant melanoma. In the A2058 xenograft model, the combination therapy had a significantly better antitumor effect compared with using dabrafenib alone. These findings were consistent with the observed in vitro synergistic effects between RC48 and dabrafenib. To guide preclinical evaluation and select the most promising ADC-based combinations for clinical use, it is crucial to deepen our understanding of ADC pharmacology and identify relevant predictive biomarkers.

To better understand how RC48, either on its own or when combined with dabrafenib, affects tumor growth, we conducted further research on their impact on the cell cycle, apoptosis, and cell motility. Our study revealed that both monotherapy and combination therapy induced apoptosis through PARP and MCL-1-dependent mechanisms. They also caused cell-cycle arrest through a CDK2 and CDK4-dependent process. Additionally, they inhibited cell motility in A2058 and SK-MEL-28 cells. These findings align with previous studies on ADC using T-DM1 and rituximab-MMAE, which also induce apoptosis and cell cycle arrest [40, 58, 59]. Therefore, the combination of RC48 and dabrafenib not only suppressed tumor cell growth by inducing apoptosis but also inhibited cell migration and invasion, ultimately leading to melanoma cell death.

In addition, our bulk RNA-seq data analysis provided insights into the molecular pathways affected by the combination therapy of RC48 and dabrafenib. The analysis revealed that this combination primarily affected genes related to PI3K-AKT pathway, MAPK pathway, AMPK pathway, FOXO pathway, p53 signaling pathway, and focal adhesion. These findings were consistent with the observed down-regulation of phosphorylation levels of AKT, mTOR, ERK, P38, AMPK, and FOXO by the combination therapy. Additionally, the combination treatment led to reduced protein levels of apoptosis-related markers (Mcl-1) and inhibited the expression of epithelial–mesenchymal transition (EMT) proteins (N-cadherin, ZEB1, and SNAI1). Furthermore, gene set enrichment analysis identified important gene sets regulated by the combination therapy, including hallmark_E2F_targets and hallmark_MYC_targets_V1, which are involved in cell cycle progression and proliferation. It is interesting to note that the overexpression of ten genes (*AIMP2*, *AURKA*, *AURKB*, *CDC45*, *CDCA3*, *CKS1B*, *HMGB3*, *KPNA2*, *MCM5*, and *NCAPD2*) and the low expression of three genes (*NUPR1*, and *SLC1A3*) were associated with poor prognosis in melanoma. These genes have been implicated in tumor cell growth, proliferation, invasion and metastasis in various cancers [60–65].

While our study provides valuable insights into the therapeutic potential of combining RC48 with dabrafenib in HER2-positive and BRAF-mutant melanoma, there are several limitations that should be acknowledged. Firstly, the low expression of HER2 in melanoma restricts the use of HER2-targeted therapies in this cancer type. Secondly, additional studies are needed to compare the effectiveness of RC48 as a standalone treatment in BRAF-mutant and wild-type melanoma. Thirdly, it is important to consider the lack of HER2 amplification or mutation models in BRAF-mutant melanoma and the absence of a direct comparison between RC48, T-DM1, and T-DXd in both in vitro and in vivo models. Lastly, further exploration is required to investigate the differentially expressed genes related to overexpression and poor prognosis in cutaneous melanoma. It is important to address these limitations in future research.

## Conclusions

Our study provides evidence supporting the therapeutic potential of the anti-HER2 drug conjugate RC48, either alone or in combination with dabrafenib, for treating HER2-positive and BRAF-mutant melanoma. Despite the generally low expression of HER2 in melanoma, our findings suggest that targeted approaches, such as ADCs, can still yield promising results. These results enlighten the possibility of overcoming challenges presented by low expression of HER2 in melanoma and offer insights into effective treatment options for this aggressive form of skin cancer. Further preclinical and clinical investigations are necessary to validate and optimize HER2-targeting therapies in melanoma, given the complexity of HER2 signaling in this cancer type. By enhancing our understanding of targeted therapies and their combinations, our goal is to improve the prognosis and long-term survival of patients with HER2-positive and BRAF-mutant cutaneous melanoma.

### Supplementary Information

**Additional file 1: Table S1.** List of antibodies used for immunofluorescence, immunohistochemistry, and western blot analysis. **Table S2.** Gene set enrichment analysis (GSEA) analysis. **Figure S1. **Internalization in cutaneous melanoma cells. The internalization and lysosomal localization of RC48 in the A2058 and SK-MEL-28 cells by confocal laser scanning microscope. The cells were treated with 2.0 μg/mL Oba01 at 4 °C for 2 h, then incubated for 0 h, 2 h, and 24 h in medium at 37 °C. The lysosomes were labeled with a LAMP-1 antibody followed by an Alexa Fluor 568-labeled goat anti-rabbit IgG (H + L) antibody. The cell nuclei were stained with Hoechst 33342. **Figure S2. **In vitro cytotoxicity of trastuzumab. A2058 and SK-MEL-28 Cells were treated with trastuzumab in indicated concentrations, and cell confluency (%) was calculated using Incucyte S3 Zoom software based on phase contrast images from 0 to 72 h. Each data point represents triplicate wells. **Figure S3. **Combined therapy of RC48 and dabrafenib significantly regulated the PI3K-AKT, MAPK, p53, Hippo, AMPK, and focal adhesion pathway DEGs expression in A2058 cells. Heatmap of significantly regulated genes of transcriptomes in A2058 cells treated with the combination of RC48 and dabrafenib (COM), correlated with the PI3K-AKT, MAPK, p53, Hippo, AMPK, and focal adhesion pathway. **Figure S4. **Expression of genes in melanoma cohort. The expression of *AIMP2*, *AURKA*, *AURKB*, *CDC45*, *CDCA3*, *CKS1B*, *HMGB3*, *KPNA2*, *MCM5*, and *NCAPD2* genes were high in melanoma in comparison to normal controls. The expression of *MAFB*, *NUPR1*, and *SLC1A3* genes were low in melanoma in comparison to normal controls. *p* < 0.05 is considered significant and was calculated by the two tailed Student’s *t*-test.

## Data Availability

All data in our study are available upon reasonable request.

## Abbreviations

HER2: Human epidermal growth factor receptor-2
ADC: Antibody–drug conjugate
MMAE: Monomethyl auristatin E
DM1: Maytansoinoid emtansine
ICIs: Immune checkpoint inhibitors
CTLA-4: Cytotoxic T lymphocyte-associated antigen-4
PD-1: Programmed death 1
MPK: mg/kg
MIR: Maximum-dose inhibitory rate
CDX: Cell-derived xenograft
OS: Overall survival
TGI: The inhibition rate of tumor growth
QW: Quaque week
H&E: Hematoxylin and eosin
IHC: Immunohistochemistry
VEH: Vehicle
DAB: Dabrafenib
FITC: Fluorescein 5-isothiocyanate
PI: Propidium iodide
PMSF: Phenylmethanesulfonyl fluoride
SDS–PAGE: Sodium dodecyl sulfate polyacrylamide gel electrophoresis
PVDF: Polyvinylidene fluoride
EdU: 5-Ethynyl-2′-deoxyuridine
ECL: Enhanced chemiluminescence
