# Supplementary material for: Combined therapy of dabrafenib and an anti-HER2 antibody–drug conjugate for advanced BRAF-mutant melanoma
Source: Cell Mol Biol Lett. 2024 Apr 10;29:50. doi: 10.1186/s11658-024-00555-z (PMC11005275; doi:10.1186/s11658-024-00555-z)
Supplement: Supplementary file 1 — Additional file 1: Table S1. List of antibodies used for immunofluorescence, immunohistochemistry, and western blot analysis. Table S2. Gene set enrichment analysis (GSEA) analysis. Figure S1. Internalization in cutaneous melanoma cells. The internalization and lysosomal localization of RC48 in the A2058 and SK-MEL-28 cells by confocal laser scanning microscope. The cells were treated with 2.0 μg/mL Oba01 at 4 °C for 2 h, then incubated for 0 h, 2 h, and 24 h in medium at 37 °C. The lysosomes were labeled with a LAMP-1 antibody followed by an Alexa Fluor 568-labeled goat anti-rabbit IgG (H + L) antibody. The cell nuclei were stained with Hoechst 33342. Figure S2. In vitro cytotoxicity of trastuzumab. A2058 and SK-MEL-28 Cells were treated with trastuzumab in indicated concentrations, and cell confluency (%) was calculated using Incucyte S3 Zoom software based on phase contrast images from 0 to 72 h. Each data point represents triplicate wells. Figure S3. Combined therapy of RC48 and dabrafenib significantly regulated the PI3K-AKT, MAPK, p53, Hippo, AMPK, and focal adhesion pathway DEGs expression in A2058 cells. Heatmap of significantly regulated genes of transcriptomes in A2058 cells treated with the combination of RC48 and dabrafenib (COM), correlated with the PI3K-AKT, MAPK, p53, Hippo, AMPK, and focal adhesion pathway. Figure S4. Expression of genes in melanoma cohort. The expression of AIMP2, AURKA, AURKB, CDC45, CDCA3, CKS1B, HMGB3, KPNA2, MCM5, and NCAPD2 genes were high in melanoma in comparison to normal controls. The expression of MAFB, NUPR1, and SLC1A3 genes were low in melanoma in comparison to normal controls. p < 0.05 is considered significant and was calculated by the two tailed Student’s t-test. [file 11658_2024_555_MOESM1_ESM.docx]

# Additional file 1

# Table S1. List of antibodies used for immunofluorescence, immunohistochemistry and western blot analysis.

| Target | Company | Cat No. | Application | Dilution |
| --- | --- | --- | --- | --- |
| HER2 | Proteintech | 18299-1-AP | IF/WB | 1：500 |
| AKT | CST | 9272 | WB | 1：2000 |
| Phospho-Akt (Ser473) | CST | 4060 | WB | 1：2000 |
| mTOR | Proteintech | 28273-1-AP | WB | 1：2000 |
| Phospho-mTOR (Ser2448) | CST | 2971 | WB | 1：2000 |
| p44/42 MAPK (Erk1/2) (137F5) | CST | 4695 | WB | 1：2000 |
| Phospho-p44/42 MAPK (Erk1/2) (Thr202/Tyr204) | CST | 4370 | WB | 1：2000 |
| p38 MAPK (D13E1) | CST | 8690 | WB | 1：2000 |
| Phospho-p38 MAPK (Thr180/Tyr182) (D3F9) | CST | 4511 | WB | 1：2000 |
| FoxO1 (C29H4) | CST | 2880 | WB | 1：2000 |
| FoxO3A | Proteintech | 10849-1-AP | WB | 1：2000 |
| Phospho-FOXO3A (Ser253) | Affinity | AF3020 | WB | 1：2000 |
| CDK2 | Affinity | AF6237 | WB | 1：2000 |
| CDK4 | Proteintech | 11026-1-AP | WB | 1：2000 |
| PARP | CST | 9532 | WB | 1：2000 |
| MCL-1 | CST | 94296 | WB | 1：2000 |
| SNAI1 | Proteintech | 13099-1-AP | WB | 1：2000 |
| ZEB1 | Proteintech | 21544-1-AP | WB | 1：2000 |
| N-cadherin | Proteintech | 22018-1-AP | WB | 1：2000 |
| Ki-67 (D2H10) Rabbit mAb (IHC Specific) | CST | 9027 | IHC | 1：500 |
| GAPDH | CST | 5174 | WB | 1：10000 |
| HRP-conjugated Affinipure Goat Anti-Rabbit IgG (H+L) | Proteintech | SA00001-2 | WB | 1：10000 |
| HRP-conjugated Affinipure Goat Anti-Mouse IgG (H+L) | Proteintech | SA00001-1 | WB | 1：10000 |

**Table S2.** Gene set enrichment analysis (GSEA) analysis

| Gene set name | NES | FDR q VALUE |
| --- | --- | --- |
| [UNFOLDED_PROTEIN_RESPONSE](http://www.gsea-msigdb.org/gsea/msigdb/human/geneset/HALLMARK_CHOLESTEROL_HOMEOSTASIS) | 2.06 | 0.000 |
| PANCREAS_BETA_CELLS | 1.69 | 0.009 |
| MYC_TARGETS_V1 | -2.04 | 0.003 |
| IL2_STAT5_SIGNALING | -1.95 | 0.003 |
| KRAS_SIGNALING_UP | -1.93 | 0.002 |
| CHOLESTEROL_HOMEOSTASIS | -1.90 | 0.002 |
| INFLAMMATOY_RESPONSE | -1.89 | 0.002 |
| GLYCOLYSIS | -1.86 | 0.002 |
| HEDGEHOG_SIGNALING | -1.85 | 0.002 |
| ANGIOGENESIS | -1.84 | 0.001 |
| COAGULATION | -1.75 | 0.004 |
| MYC_TARGETS_V2 | -1.72 | 0.005 |
| EPITHELIAL_MESENCHYMAL_  TRANSITION | -1.67 | 0.007 |
| HYPOXIA | -1.64 | 0.009 |
| FATTY_ACID_METABOLISM | -1.56 | 0.019 |
| OXIDATIVE_PHOSPHORYLATION | -1.56 | 0.018 |
| NOTCH_SIGNALING | -1.55 | 0.026 |
| MTORC1_SIGNALING | -1.53 | 0.002 |
| E2F_SIGNALING | -1.53 | 0.019 |
| UV_RESPONSE_DN | -1.48 | 0.027 |
| ANDROGEN_RESPONSE | -1.46 | 0.030 |
| ESTROGEN_RESPONSE_EARLY | -1.43 | 0.036 |


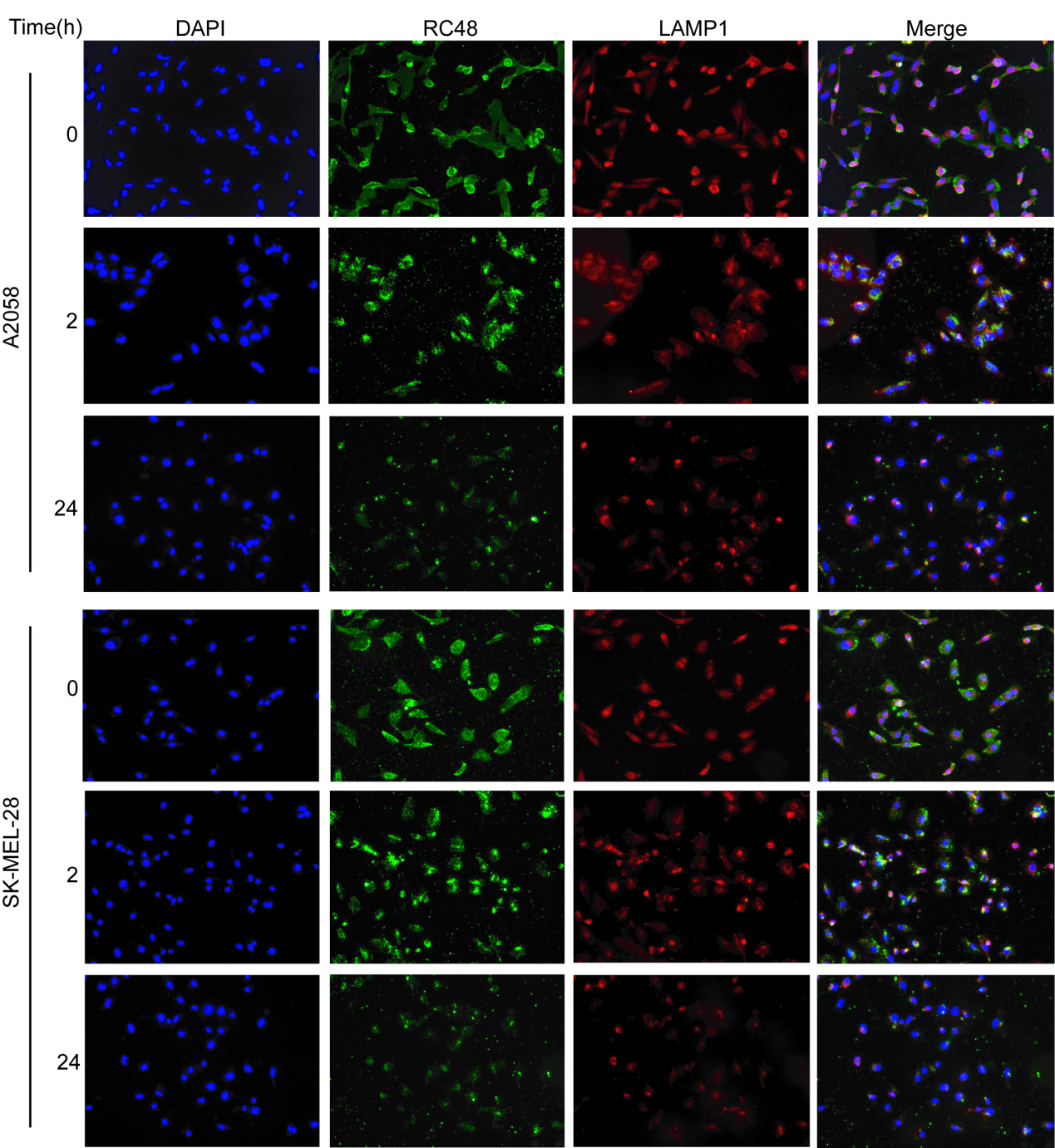


**Figure S1. Internalization in cutaneous melanoma cells.** The internalization and lysosomal localization of RC48 in the A2058 and SK-MEL-28 cells by confocal laser scanning microscope. The cells were treated with 2.0 μg/mL Oba01 at 4 °C for 2h, then incubated for 0, 2h and 24h in medium at 37°C. The lysosomes were labeled with a LAMP-1 antibody followed by an Alexa Fluor 568-labeled goat anti-rabbit IgG (H+L) antibody. The cell nuclei were stained with Hoechst 33342.

**
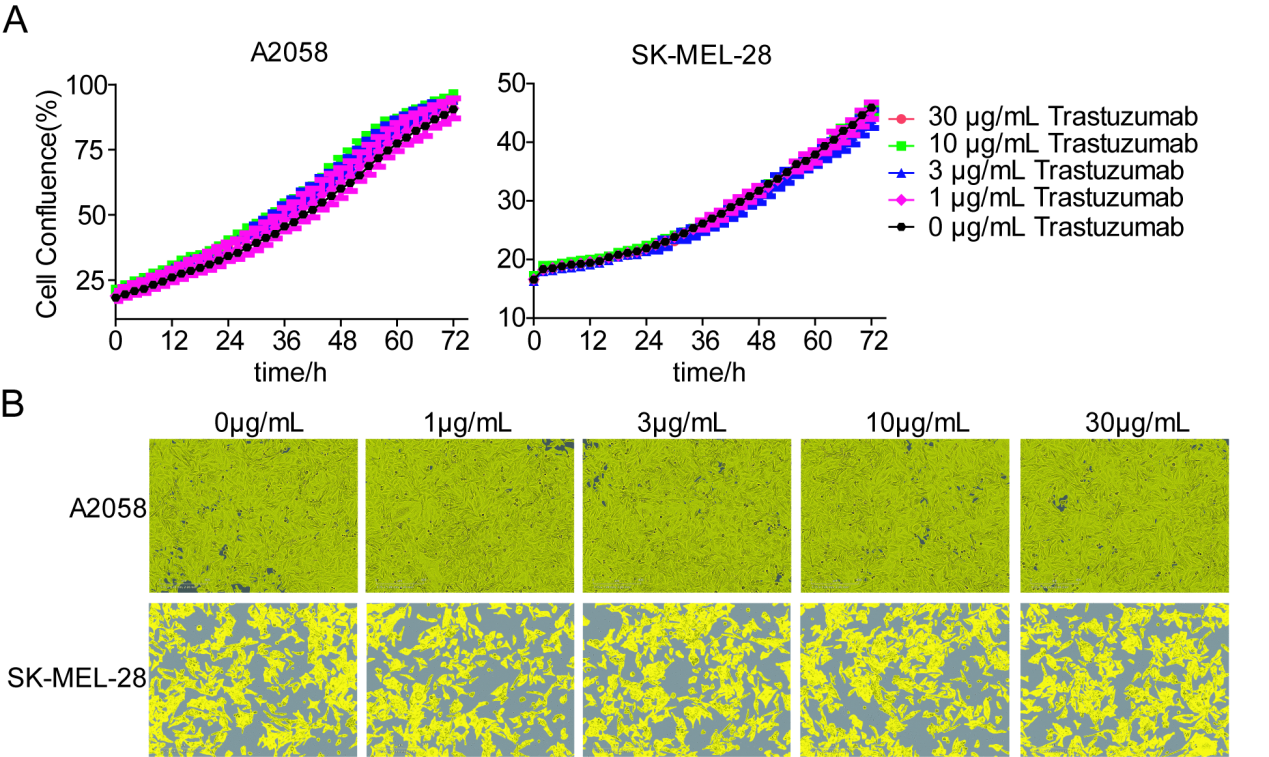
Figure S2.** *In vitro* cytotoxicity of trastuzumab**.** A2058 and SK-MEL-28 Cells were treated with trastuzumab in indicated concentrations, Cell confluency (%) was calculated using Incucyte S3 Zoom software based on phase-contrast images from 0 h to 72 h. Each data point represents triplicate wells.


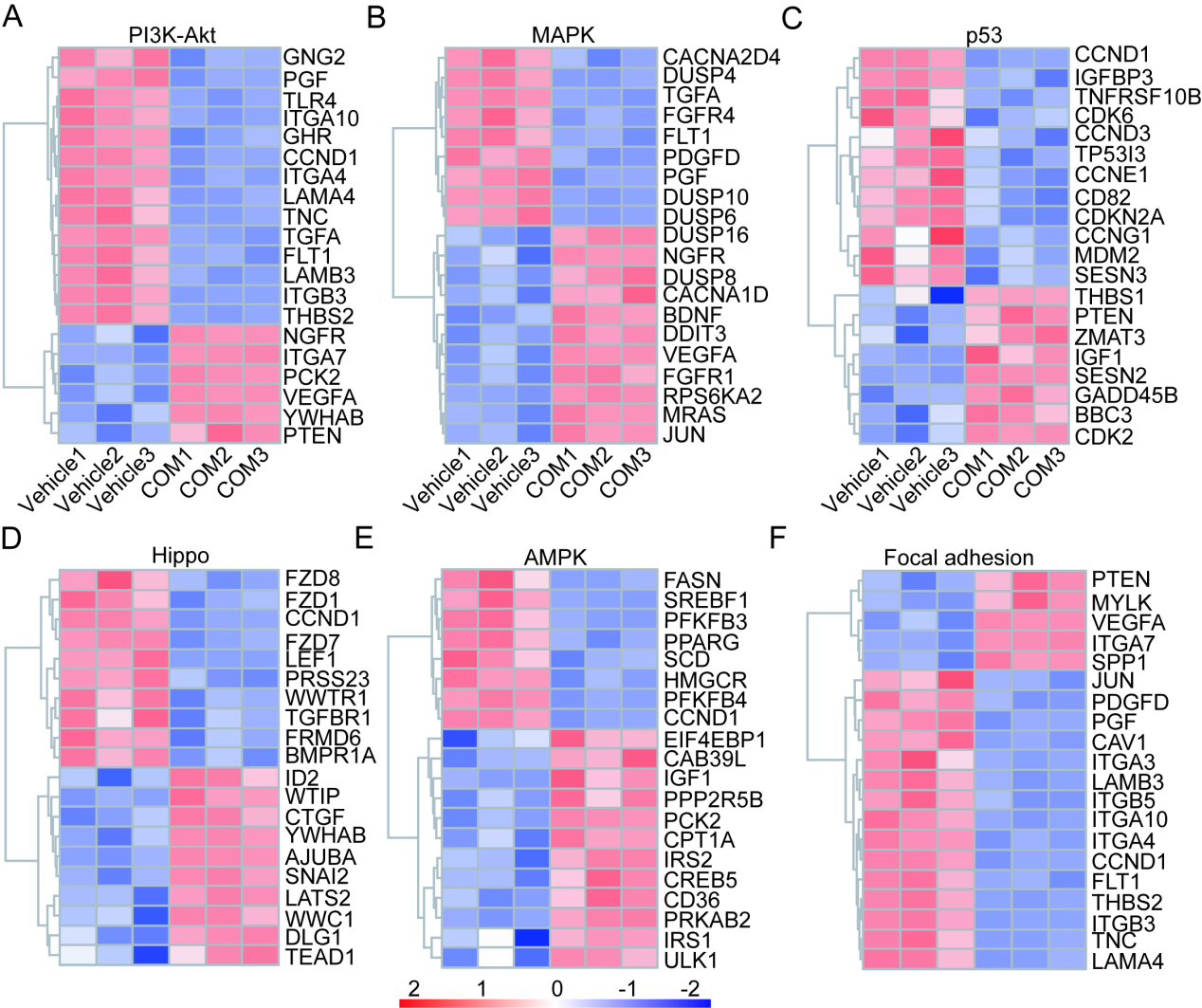


**Figure S3. Combined therapy of RC48 and dabrafenib significantly regulated the PI3K-AKT, MAPK, p53, Hippo, AMPK and Focal adhesion pathway DEGs expression in A2058 cells.** Heatmap of significantly regulated genes of transcriptomes in A2058 cells treated with the combination of RC48 and dabrafenib (COM), correlated with the PI3K-AKT, MAPK, p53, Hippo, AMPK and Focal adhesion pathway.

.


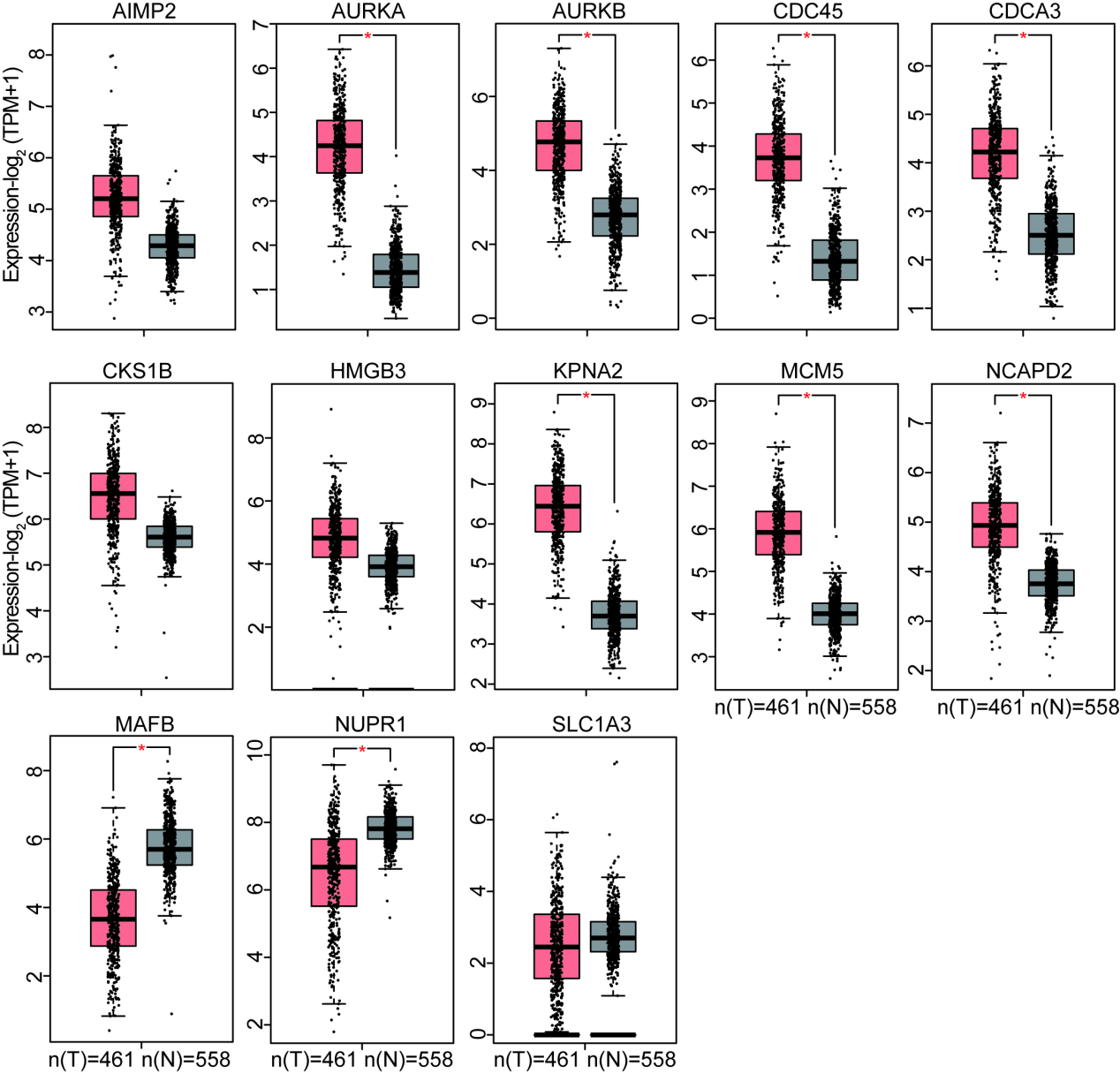


**Figure S4. Expression of genes in melanoma cohort.** The expression of AIMP2, AURKA, AURKB, CDC45, CDCA3, CKS1B, HMGB3, KPNA2, MCM5 and NCAPD2 genes were high in melanoma in comparison to normal controls. The expression of MAFB, NUPR1 and SLC1A3 genes were low in melanoma in comparison to normal controls. p<0.05 is considered significant and was calculated by the two tailed Student’s t test.
